# Arterial Thrombosis in Severe Ulcerative Colitis: A Case-Based Narrative Review of Current Evidence

**DOI:** 10.3390/biomedicines14030559

**Published:** 2026-02-28

**Authors:** Djordje Kralj, Mladen Maksic, Tamara Knezevic Ivanovski, Olga Odanovic, Tijana Maksic, Tijana Gmizic, Zeljko Ivosevic, Dusan Radojevic, Lejla Suljic, Nevena Todorovic, Natasa Zdravkovic, Irfan Corovic, Srdjan Markovic

**Affiliations:** 1 Department of Gastroenterology, University Hospital Medical Center Zvezdara, 11000 Belgrade, Serbia; drkraljdjordje@gmail.com (D.K.); tamara6788@gmail.com (T.K.I.); olga.odanovic@gmail.com (O.O.); 2 Department of Internal Medicine, Faculty of Medical Sciences, University of Kragujevac, Svetozara Markovica 69, 34000 Kragujevac, Serbia; asussonicmaster95@gmail.com (M.M.); zeljkoivosevic274@gmail.com (Z.I.); radojevicdusan@yahoo.com (D.R.); natasasilvester@gmail.com (N.Z.); 3 Clinic of Gastroenterology and Hepatology, University Clinical Centre Kragujevac, Zmaj Jovina 30, 34000 Kragujevac, Serbia; 4 Faculty of Medicine, University of Belgrade, Pasterova 2, 11000 Belgrade, Serbia; 5 Department of Pediatrics, Faculty of Medical Sciences, University of Kragujevac, Svetozara Markovica 69, 34000 Kragujevac, Serbia; tijanaveljkovic96@gmail.com; 6 Pediatric Clinic, University Clinical Centre Kragujevac, Zmaj Jovina 30, 34000 Kragujevac, Serbia; 7 Department of Gastroenterology and Hepatology, Clinic of Internal Medicine, University Hospital Medical Center Bežanijska Kosa, Dr Žorža Matea bb, 11000 Belgrade, Serbia; gmizic.tijana@bkosa.edu.rs; 8 Clinic for Endocrinology, Diabetes and Metabolic Diseases, University Clinical Center Kragujevac, Zmaj Jovina 30, 34000 Kragujevac, Serbia; 9 Center for Molecular Medicine and Stem Cell Research, Faculty of Medical Sciences, University of Kragujevac, Svetozara Markovica 69, 34000 Kragujevac, Serbia; lejlasuljic990@gmail.com; 10 Department of Experimental and Clinical Medicine, University of Florence, 50134 Florence, Italy; nevena.todorovic@unifi.it; 11 Clinic for Infectious and Tropical Diseases, University Clinical Centre of Serbia, 11000 Belgrade, Serbia; 12 Department of Internal Medicine, General Hospital of Novi Pazar, Generala Zivkovica 1, 36300 Novi Pazar, Serbia

**Keywords:** arterial thrombosis, inflammatory bowel disease, IBD, inflammation, ulcerative colitis, ischemic stroke

## Abstract

Inflammatory bowel disease is a recognized risk factor for venous thromboembolism, whereas arterial thrombotic events remain underappreciated despite their substantial clinical consequences. We report a 45-year-old man without significant comorbidities who developed severe ulcerative colitis complicated by diffuse arterial thrombosis, including cerebral infarctions, an ascending aortic mural thrombus, iliac artery thrombosis, and multi-organ infarctions. After stabilization with supportive care and anticoagulation, remission-directed ulcerative colitis therapy and a vascular safety–oriented maintenance strategy were initiated, including vedolizumab and individualized secondary thrombosis prevention. To contextualize this presentation, we integrate current evidence on the epidemiology, clinical phenotypes, underlying mechanisms, and risk factors for arterial thrombosis in inflammatory bowel disease, highlight disease activity as a dominant trigger, and summarize therapy-specific vascular safety considerations across IBD treatment classes.

## 1. Introduction

Inflammatory bowel disease (IBD) is a chronic immune-mediated disorder associated with a substantially increased risk of thromboembolic complications, most prominently venous thromboembolism (VTE) [1,2,3]. In contrast, arterial thromboembolic events have received considerably less attention. Although less frequent than venous events, arterial thromboses are associated with severe and potentially life-threatening consequences [4,5,6]. Emerging data indicate that patients with IBD are at increased risk for such arterial events; however, these complications remain underrecognized and incompletely characterized in both clinical practice and the literature.

The prothrombotic milieu in IBD is multifactorial, arising from a complex interplay between systemic inflammation, endothelial dysfunction, platelet activation, and dysregulation of coagulation and fibrinolytic pathways [2,7,8]. Notably, arterial thrombosis may develop even in the absence of traditional cardiovascular risk factors, particularly in the setting of uncontrolled inflammation during active disease [3,4,5,6,9].

Therefore, we present a case of diffuse arterial thromboembolism in a young patient with active ulcerative colitis and no conventional cardiovascular risk factors. Additionally, we conducted a comprehensive literature review on the epidemiology, clinical manifestations, pathophysiological mechanisms, risk factors, and differential diagnosis of arterial thrombosis in IBD, followed by an evidence-based clinical discussion of the present case and its implications for future research and clinical practice.

## 2. Case Presentation

### 2.1. History and Presentation

A 45-year-old man presented to the Emergency Department of the University Clinical Center of Kragujevac with malaise, abdominal pain, tenesmus, urgency, and bloody stools. Symptoms had been present for 18 months, gradually worsening, with significant deterioration during the preceding week (>6 bloody stools per day). He denied prior illnesses, medication or food allergies, smoking, alcohol, or illicit drug use.

### 2.2. Examination and Initial Workup

On admission (Day 0), his vital signs were stable. Physical examination revealed pallor of the skin and conjunctiva and positive blood on digital rectal examination. The remainder of the systemic exam was unremarkable. Laboratory analysis showed iron-deficiency anemia (hemoglobin 117 g/L), leukocytosis with neutrophilia, thrombocytosis, and elevated inflammatory markers (C-reactive protein, D-dimer, fibrinogen). Coagulation tests (PT, aPTT), renal and hepatic function, electrolytes, and tumor markers were within reference limits (Table 1).

Microbiological testing, including PCR stool panel, was negative. Chest radiography, abdominal ultrasound, and upper endoscopy were unremarkable. Flexible rectosigmoidoscopy revealed continuous severe inflammation of the rectum and sigmoid colon, with fibrin-covered ulcerations and contact bleeding (Mayo endoscopic subscore 3). Biopsies confirmed UC.

### 2.3. Initial Treatment

Therapy was initiated with intravenous methylprednisolone (60 mg OD), high-dose oral mesalazine (2 g BID), prophylactic nadroparin, pantoprazole, probiotics, intravenous fluids, and intravenous metronidazole (500 mg TID).

### 2.4. Neurological and Thrombotic Complications

**Figure 1:**
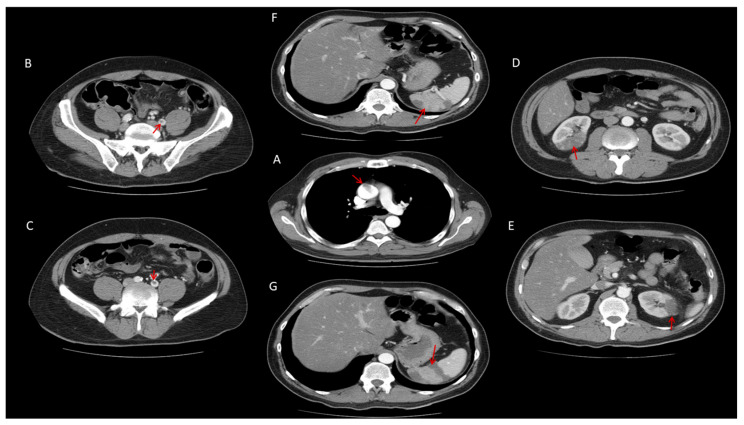
Contrast-enhanced CT of the thorax and abdomen (Day 4). (**A**) Mural thrombus in the ascending aorta. (**B**,**C**) Filling defects in the left iliac artery consistent with arterial thrombi. (**D**,**E**) Infarct zones in both kidneys. (**F**,**G**) Infarct zones in the liver and spleen.

On Day 3, the patient developed acute neurological symptoms, including dysarthria and right-sided hemiparesis. Initial non-contrast brain CT was unremarkable. On Day 4, repeat contrast-enhanced CT demonstrated a 15 mm hypodensity in the left insular region consistent with acute ischemia, and a minor lesion in the left corona radiata. In search of etiology, CT of the thorax, abdomen, and pelvis (Day 4) revealed mural thrombus in the ascending aorta (15 mm), thrombi in the left iliac artery, and multiple infarct zones in the liver, spleen, and kidneys (Figure 1).

**Figure 2:**
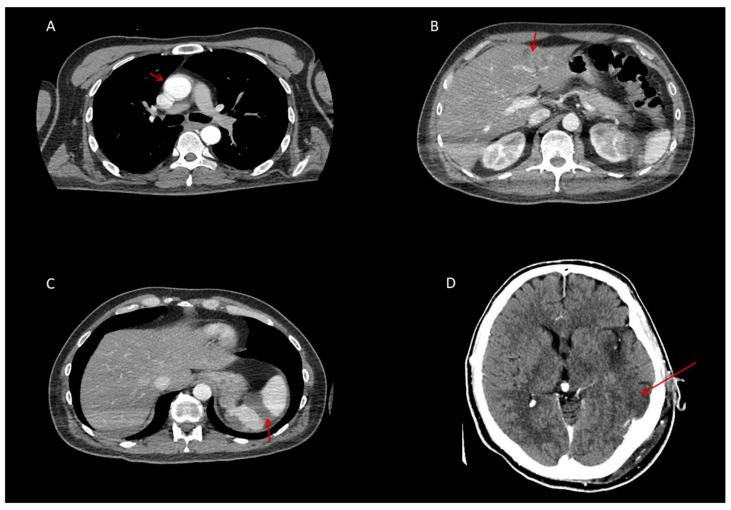
Contrast-enhanced CT and brain imaging (Day 9): (**A**) abdominal aorta; (**B**,**C**) infarct zones in the liver and spleen; (**D**) left occipito-temporo-parietal ischemic lesion with additional subacute infarctions.

The patient’s condition rapidly deteriorated with confusion, requiring intubation and mechanical ventilation. Anticoagulation was escalated to unfractionated heparin (500 IU/kg), titrated to maintain an aPTT of 75–90 s, while nadroparin was discontinued. Follow-up whole-body CT performed on Day 9 showed that the thrombi in the ascending aorta and iliac artery were no longer visible, indicating resolution (Figure 2). At the same time, infarct zones in the liver, spleen, and kidneys remained without dynamic progression (Figure 2). A control CT of the brain confirmed progression of ischemic changes, with a larger hypodense lesion in the left occipito-temporo-parietal region and four additional periventricular and subcortical hypodense areas (12–14 mm), consistent with subacute cerebrovascular infarctions (Figure 2).

Despite these findings, his clinical status gradually improved under supportive therapy and anticoagulation. He was successfully extubated on Day 12, with residual dysarthria, right hemiparesis, and central facial palsy, but remained hemodynamically stable and responsive to rehabilitation.

### 2.5. Hematological, Immunological, and Infectious Workup

A comprehensive evaluation of potential causes of arterial thrombosis was performed (Table 1). Tests for anti-cardiolipin IgG/IgM, anti-β2 glycoprotein I IgG/IgM), lupus anticoagulant, protein C and S deficiencies, antithrombin deficiency, factor V Leiden mutation, and prothrombin G20210A mutation were all negative. Genetic testing revealed homozygosity for the MTHFR C677T variant and the PAI-1 4G/5G polymorphism. Serum homocysteine was mildly elevated. Screening for autoimmune conditions, including RF (reuma factor), anti-CCP (anti-cyclic citrulinated peptide), anti-GBM (anti-glomerular basement membrane antibodies), antinuclear antibodies (ANA), anti-neutrophil cytoplasmic antibodies (cANCA and pANCA) was negative, as well as tumor markers. Serologic testing for viral infections, including hepatitis B virus (HBV), hepatitis C virus (HCV), and human immunodeficiency virus (HIV), and SARS-CoV-2 (severe acute respiratory syndrome Coronavirus 2) was also negative.

### 2.6. Outcome

After induction of remission, corticosteroids were tapered and vedolizumab (ENTYVIO^®^, Takeda Pharmaceuticals, Deerfield, IL, USA) was introduced as maintenance therapy. Secondary prophylaxis with clopidogrel (75 mg/day) was initiated, as well as folic acid, vitamin B6, B12, and liposomal iron-pyrophosphate supplementation. At 6-month follow-up, the patient remained in clinical, biochemical, and endoscopic remission of UC, with no recurrent thrombotic events or neurological sequelae.

## 3. Discussion

### 3.1. Arterial Thrombosis in IBD: Epidemiology and Clinical Phenotypes

Patients with IBD consistently exhibit an approximately twofold increased risk of VTE compared with the general population [10,11,12]. Yuhara et al. [10] first reported a 2.2-fold higher risk of deep vein thrombosis and pulmonary embolism, findings subsequently confirmed in large population-based cohorts by Fumery et al. [11]. Notably, Kappelman et al. [12] demonstrated that this excess risk extends to younger individuals, and its persistence after adjustment for conventional thromboembolic risk factors supports IBD as an independent hypercoagulable state. Beyond VTE, accumulating evidence indicates that IBD is also associated with an increased burden of arterial thromboembolic events. Although the magnitude of risk appears more modest than for venous events, it remains clinically relevant. A spectrum of arterial outcomes, including ischemic stroke, acute coronary syndrome (ACS), peripheral artery thrombosis, and mesenteric ischemia, has been described, albeit with variable consistency across studies (Table 2).

#### 3.1.1. Ischemic Stroke

Among these, ischemic stroke has emerged as the most consistently reported arterial complication. Large population-based cohort studies and meta-analyses demonstrate a higher incidence of stroke in IBD compared with matched controls, with hazard ratios (HRs) ranging from 1.13 to 1.30 for overall stroke and from 1.14 to 1.35 for ischemic stroke specifically [13,14,15]. The excess risk appears most pronounced in CD, whereas several studies have reported no statistically significant increase in UC [15]. Importantly, this elevated risk persists for more than 25 years following IBD diagnosis [13]. Subgroup analyses suggest that women and younger patients exhibit a higher relative risk of ischemic stroke [5,16]. Clinically, ischemic strokes in IBD most frequently involve the middle cerebral artery territory and typically present with focal neurological deficits such as hemiparesis [17]. Notably, cerebrovascular events may occur in the absence of conventional cardiovascular risk factors, particularly during periods of active disease, when laboratory features of a hypercoagulable state are commonly observed [18]. In addition to arterial ischemic stroke, other cerebrovascular complications, including retinal artery occlusion, have also been reported in the context of IBD [18].

#### 3.1.2. Acute Coronary Syndrome

Epidemiological data further demonstrate that patients with IBD, encompassing both CD and UC, have a modest but clinically meaningful increased risk of ACS compared with the general population, even after adjustment for traditional cardiovascular risk factors such as hypertension, diabetes mellitus, and dyslipidemia [16,19,20,21,22]. Although the absolute incidence of ACS increases with age, the relative risk is disproportionately higher among younger IBD patients, particularly those aged 20–39 years [20]. Disease-related factors further modulate this risk, as multiple studies have shown that ACS risk is amplified during periods of active disease, frequent hospitalizations, and in the presence of extra-intestinal manifestations, underscoring the contribution of systemic inflammatory burden rather than conventional atherosclerotic risk alone [16,19,20,23]. Large population-based cohorts and meta-analyses estimate that the incidence of ACS in IBD patients is approximately 1.2- to 1.7-fold higher than in matched controls, with broadly comparable risk estimates for CD and UC [19,20,21,22]. Notably, despite a lower prevalence of classic cardiovascular risk factors, IBD patients continue to experience increased rates of ACS, reinforcing the concept that chronic inflammation and disease activity are key drivers of coronary risk in this population [16,21,22]. Clinically, the presentation of ACS in IBD largely mirrors that of the general population, with chest pain, dyspnea, and electrocardiographic changes predominating [24]. However, hospitalizations for ACS in IBD are more frequently complicated by coagulopathy, malnutrition or weight loss, and gastrointestinal bleeding, each of which independently predicts in-hospital mortality [24]. Despite these challenges, reported in-hospital mortality rates for ACS are modestly lower in IBD patients than in non-IBD ACS cohorts, potentially reflecting differences in baseline cardiovascular risk profiles, age distribution, and management strategies [24]. Importantly, rates of coronary angiography and revascularization appear comparable between IBD and non-IBD patients presenting with ACS [24].

#### 3.1.3. Peripheral Artery Thrombosis and Mesenteric Ischemia

Peripheral artery thrombosis and mesenteric ischemia further broaden the spectrum of arterial thromboembolic manifestations in IBD. Overall, population-based studies and meta-analyses suggest that the association between IBD and peripheral artery thrombosis is modest and less consistent than that observed for cerebrovascular or coronary events, with some analyses failing to reach statistical significance [5]. Nevertheless, clinically relevant peripheral artery thrombosis risk emerges in specific high-risk subgroups. In a nationwide Taiwanese cohort including more than 11,000 IBD patients, Lin et al. [25] demonstrated a 29% increased risk of peripheral artery thrombosis (HR 1.29), an association consistent across both CD and UC. Strikingly, patients requiring two or more hospitalizations per year exhibited a 27.5-fold higher risk of peripheral artery thrombosis, underscoring disease severity as a critical determinant of peripheral arterial thrombotic risk. Similarly, Bernstein et al. [26] reported an increased incidence rate ratio (IRR) for undifferentiated arterial thromboembolic disease, encompassing peripheral arterial events, among IBD patients, with the highest relative risks observed in women and younger individuals, particularly those aged 0–39 years (IRR up to 19.95), albeit with wide confidence intervals reflecting small event numbers.

In contrast to peripheral artery thrombosis, the association between IBD and mesenteric ischemia appears substantially stronger. Large cohort studies and meta-analyses consistently demonstrate a several-fold increased risk of mesenteric ischemia in patients with IBD compared with matched controls [16,20,27,28]. One nationwide cohort reported an adjusted HR of 6.33 for mesenteric ischemia, with the highest relative risk observed in patients younger than 44 years (adjusted HR 48.0) and the greatest absolute risk occurring within the first year following IBD diagnosis [20]. Similarly, a large claims-based analysis demonstrated an elevenfold increased risk of acute mesenteric ischemia among IBD patients (HR 11.2) [27]. Importantly, this excess risk is observed across both CD and UC and persists after adjustment for traditional cardiovascular risk factors [27]. Several studies further demonstrate that the risk of mesenteric ischemia is markedly increased during periods of active disease and acute flares, reflecting the profound prothrombotic and inflammatory milieu characteristic of uncontrolled IBD [16,28].

**Table 2:** Key Studies on Arterial Thrombotic Risk in IBD.

Study/Year	Design & Population	Arterial Outcomes	Key Findings
Sun J. et al., 2023 [13]	Swedish nationwide cohort; 85,006 IBD pts; matched population and sibling controls; 1969–2019	Overall, ischemic, and hemorrhagic stroke	Overall stroke aHR 1.13; ischemic stroke aHR 1.14; persistent > 25 yrs
Luo C. et al., 2025 [14]	Systematic review and meta-analysis; 13 studies; 2.8 M participants	Stroke	HR 1.30; CD HR 1.35; UC HR 1.15
Tanislav C. et al., 2021 [15]	German cohort; 11,947 IBD vs. 11,947 controls	Stroke, TIA	Stroke HR 1.30; CD-specific excess
Schneiderman J.H., 1979 [18]	Case series; 5 IBD pts	Retinal & cerebral thrombosis	Hypercoagulability; severe events
Singh S. et al., 2014 [5]	Meta-analysis; 9 studies	CVA, IHD	CVA OR 1.18; IHD OR 1.19
Eriksson C. et al., 2024 [19]	Swedish cohort; 76,517 IBD vs. 757,141 controls	ACS	HR 1.30; highest in elderly-onset, EIM
Tsai M.S. et al., 2014 [20]	Taiwanese cohort; 11,822 IBD vs. 47,288 controls	ACS	aHR 1.72; highest in age 20–39; severity-dependent
Aniwan S. et al., 2018 [21]	Population-based cohort; 736 IBD vs. 1472 controls	AMI, heart failure	AMI aHR 2.82; HF aHR 2.03; steroid users at highest risk
D’Ascenzo F. et al., 2023 [22]	Systematic review & meta-analysis; 77,140 IBD vs. 515,455 controls	MI, stroke, mortality	MI HR 1.36 (CD), 1.24 (UC); stroke HR 1.22 (CD), 1.09 (UC)
Pemmasani G. et al., 2021 [24]	U.S. NIS; 24,220 IBD-ACS vs. 6.9 M non-IBD ACS	ACS outcomes	Lower in-hospital mortality (OR 0.81); IBD complications predict death
Lin T.Y. et al., 2015 [25]	Taiwanese nationwide cohort; 11,067 IBD vs. 43,765 controls	PAD	PAD HR 1.29; severe IBD → ~27.5 × risk
Bernstein C.N. et al., 2008 [26]	Canadian cohort; 8060 IBD vs. 80,489 controls	IHD, cerebrovascular disease, ATED	IHD IRR 1.26; cerebrovascular IRR 1.32 in CD
Ha C. et al., 2009 [27]	U.S. claims cohort; 17,487 IBD vs. 69,948 controls	Mesenteric, cerebral, cardiac arterial events	Acute mesenteric ischemia HR 11.2; ↑ MI in women > 40; ↑ stroke in women < 40

**Abbreviations**: **IBD**, Inflammatory Bowel Disease; **CD**, Crohn’s Disease; **UC**, Ulcerative Colitis; **ACS**, Acute Coronary Syndrome; **AMI**, Acute Myocardial Infarction; **HF**, Heart Failure; **MI**, Myocardial Infarction; **IHD**, Ischemic Heart Disease; **CVA**, Cerebrovascular Accident; **TIA**, Transient Ischemic Attack; **PAD**, Peripheral Arterial Disease; **ATED**, Arterial Thromboembolic Disease; **HR**, Hazard Ratio; **aHR**, Adjusted Hazard Ratio; **OR**, Odds Ratio; **IRR**, Incidence Rate Ratio; **EIM**, Extra-Intestinal Manifestations; **NIS**, National Inpatient Sample.

### 3.2. Pathophysiological Mechanisms Linking IBD and Thrombosis

Inflammatory bowel disease is characterized by a dysregulated immune response to luminal antigens that penetrate the disrupted intestinal barrier, resulting in sustained activation of both innate and adaptive immunity [29,30,31,32]. Beyond driving mucosal inflammation, this exaggerated immune response directly engages the hemostatic system through an evolutionarily conserved process known as immunothrombosis, in which immune activation and coagulation are tightly interconnected [33,34,35,36]. This ancestral link between inflammation and coagulation evolved to limit microbial dissemination and tissue invasion; however, in IBD, loss of immune and hemostatic control transforms this protective mechanism into a major driver of pathological thrombosis.

Activated immune cells, including monocytes, neutrophils, and lymphocytes, modulate coagulation at multiple levels [33,34,35,36,37,38,39,40,41]. Stimulation by pathogen-associated and damage-associated molecular patterns (PAMPs and DAMPs) activates pattern recognition receptors (e.g., Toll-like receptors), leading to upregulation of tissue factor (TF) on leukocytes and endothelial cells and the release of TF-bearing extracellular vesicles, thereby initiating and amplifying thrombin generation [33,34,35,36,37]. In parallel, activated neutrophils and monocytes release granular enzymes, cytokines, and DAMPs, such as histones, high-mobility group box 1 (HMGB1), and nucleic acids, which further propagate both inflammation and coagulation. These DAMPs can directly activate coagulation factors and impair endogenous anticoagulant pathways, contributing to a self-perpetuating cycle of immunothrombosis [34,36,37]. Furthermore, activated neutrophils form neutrophil extracellular traps (NETs) composed of DNA, histones, and proteases. NETs provide a scaffold for platelet adhesion and activation, concentrate TF, and directly activate factor XII, thereby linking innate immune responses to both intrinsic and extrinsic coagulation pathways [34,36,37,38]. Chemokines released by immune cells further modulate thrombosis by regulating platelet activation, endothelial adhesiveness, and leukocyte recruitment to sites of vascular injury. Certain chemokines, such as CXCL12, can directly activate platelets and promote aggregation through specific signaling pathways, independent of classical chemokine receptor activity [38]. Adaptive immune cells also contribute to this process. CD4^+^ T cells, particularly Th17 subsets, are recruited to sites of fibrin deposition and can be activated through direct interactions with fibrin matrices. Activated T cells limit excessive fibrin accumulation and microvascular thrombosis by enhancing fibrinolysis via lymphocyte function–associated antigen 1 (LFA-1)-dependent mechanisms and by inhibiting the association of thrombin-activatable fibrinolysis inhibitor (TAFI) with fibrin, thereby establishing a negative feedback loop that restrains pathological thrombosis while linking chronic intestinal inflammation to systemic vascular risk [40].

Consistent with this immune-driven hemostatic activation, the coagulation cascade is profoundly dysregulated in IBD. Elevated fibrinogen levels, increased activity of coagulation factors V, VII, VIII, XI, and von Willebrand factor, and heightened thrombin generation markers (F1+2, TAT complexes, D-dimers) reflect a persistent hypercoagulable state that is further amplified by TF overexpression and reduced factor XIII activity [1,23,24]. At the same time, endogenous anticoagulant pathways, including protein C, protein S, antithrombin III, and tissue factor pathway inhibitor, are frequently impaired, while hypofibrinolysis driven by reduced tissue plasminogen activator and increased PAI-1 and TAFI promotes fibrin persistence, reinforcing the inflammatory-coagulant loop [1,41,42].

Platelet abnormalities represent an additional critical component of immunothrombosis in IBD. Thrombocytosis correlates with disease activity, whereas mean platelet volume is inversely associated with inflammatory markers such as C-reactive protein and erythrocyte sedimentation rate [24]. Platelets exhibit chronic activation, enhanced spontaneous aggregation, and increased expression of P-selectin, β-thromboglobulin, and CD40/CD40L, alterations that may persist even during clinical remission [2]. Through CD40/CD40L signaling, activated platelets act as inflammatory effector cells, promoting leukocyte recruitment, endothelial activation, microvascular thrombosis, and mucosal inflammation [2,42]. Clinically, persistent thrombocytosis has been linked to increased cardiovascular risk, and meta-analyses consistently demonstrate higher platelet counts and lower mean platelet volume in patients with IBD [43,44].

Endothelial dysfunction constitutes a central pathogenic hub in this process. Under the influence of pro-inflammatory cytokines, endotoxins, hypoxia, and altered shear stress, the normally anticoagulant endothelium adopts a prothrombotic phenotype characterized by increased expression of adhesion molecules, loss of antithrombotic surface properties, and enhanced leukocyte adhesion. CD40/CD40L interactions further amplify endothelial activation, establishing a self-perpetuating cycle supported by histological evidence of mucosal microthrombi and elevated circulating endothelial markers. These observations form the basis of the vascular hypothesis of IBD, linking chronic intestinal inflammation, systemic hypercoagulability, microvascular injury, and accelerated atherosclerosis [1].

Importantly, IBD shares mechanistic pathways with atherogenesis. Chronic exposure to inflammatory cytokines such as TNF-α, IL-6, and VEGF, disruption of the intestinal barrier with translocation of microbial products, and Toll-like receptor signaling promote oxidative stress, endothelial injury, and plaque instability. Activated Th1 cells and interferon-γ further contribute to vascular inflammation and plaque destabilization, underscoring the overlap between intestinal inflammation, immunothrombosis, and increased cardiovascular risk in IBD [45].

**Figure 3:**
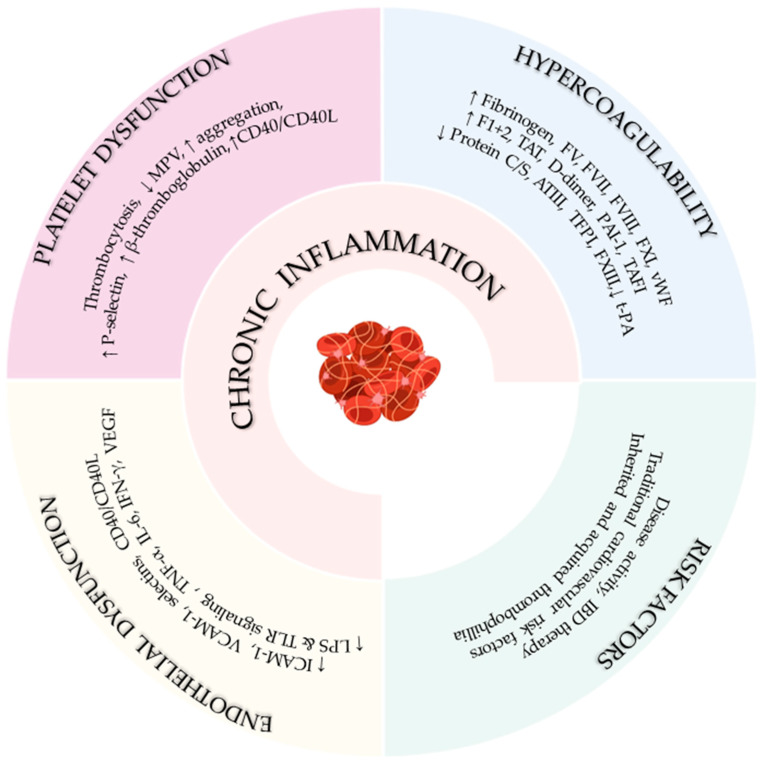
Pathophysiological mechanisms contributing to arterial thrombosis in IBD. **Abbreviations**: **FV**, coagulation factor V; **FVII**, coagulation factor VII; **FVIII**, coagulation factor VIII; **FXI**, coagulation factor XI; **vWF**, von Willebrand factor; **F1+2**, prothrombin fragment 1+2; **TAT**, thrombin-antithrombin complex; **FXIII**, coagulation factor XIII; **ATIII**, antithrombin III; **TFPI**, tissue factor pathway inhibitor; **t-PA**, tissue plasminogen activator; **PAI-1**, plasminogen activator inhibitor-1; **TAFI**, thrombin-activatable fibrinolysis inhibitor; **CD40L**, CD40 ligand; **ICAM-1**, intercellular adhesion molecule-1; **VCAM-1**, vascular cell adhesion molecule-1; **LPS**, lipopolysaccharide; **TLR**, toll-like receptor; **TNF-α**, tumor necrosis factor-alpha; **IL-6**, interleukin-6; **IFN-γ**, interferon-gamma; **VEGF**, vascular endothelial growth factor.

Overall, thrombotic risk in IBD reflects a multifactorial, predominantly acquired process driven by immune-mediated hypercoagulability, platelet activation, endothelial dysfunction, and altered blood flow (Figure 3). In contrast, inherited thrombophilias play a limited and inconsistent role [1,3]. The convergence of dysregulated immunity and coagulation, hallmarks of inflammation-driven immunothrombosis, provides a unifying mechanistic framework for the markedly increased thromboembolic burden observed in both individual patients and the broader IBD population (Figure 1).

### 3.3. Triggers and Modifiers of Arterial Thrombotic Risk in IBD

#### 3.3.1. Disease Activity

Active IBD is independently associated with an increased risk of arterial thrombotic events, including ACS and ischemic stroke. Large population-based cohort studies and meta-analyses consistently demonstrate that the incidence of acute arterial events is significantly higher during periods of clinical or histologic disease activity, whereas this excess risk attenuates and approaches baseline during remission [46,47,48,49,50]. Nationwide registry data from Denmark and France, for example, indicate that the risk of myocardial infarction, stroke, and cardiovascular mortality peaks during IBD flares or sustained inflammatory activity, with rate ratios for arterial events increasing by up to twofold compared with remission or the general population [46,50]. Importantly, these associations persist after adjustment for traditional cardiovascular risk factors, supporting the concept that IBD activity itself constitutes an independent determinant of arterial thrombotic risk [47,48]. Le Gall et al. [48] demonstrated that both clinical disease activity (odds ratio [OR] 10.4) and systemic inflammation, as reflected by elevated C-reactive protein levels (≥5 mg/L), independently predicted acute arterial events in patients with IBD. The magnitude of risk appears particularly pronounced in younger patients and during the first year following IBD diagnosis, likely reflecting a heightened inflammatory burden during early disease [16,28,50]. Both clinical and histologic disease activity confer excess arterial risk, and notably, even subclinical inflammation, defined by histologic activity in the absence of overt clinical symptoms, has been associated with increased rates of myocardial infarction and heart failure [49].

Emerging evidence further suggests that effective suppression of intestinal inflammation through appropriate medical therapy may mitigate arterial thrombotic risk [16]. Collectively, these findings underscore the critical importance of achieving and maintaining tight inflammatory control in IBD, as well as incorporating cardiovascular risk assessment into routine disease management, particularly during periods of active inflammation [16,28,51].

#### 3.3.2. Traditional Cardiovascular Risk Factors

Traditional cardiovascular risk factors, including hypertension, diabetes mellitus, hyperlipidemia, smoking, and obesity, contribute to arterial thrombosis through mechanisms that promote atherosclerosis, endothelial dysfunction, and a prothrombotic state, increasing the risk of myocardial infarction, ischemic stroke, and peripheral arterial thrombosis [52,53,54]. Hypertension and diabetes mellitus promote endothelial injury, platelet activation, and increased thrombogenicity, particularly when metabolic control is poor [52,53], while hyperlipidemia, especially elevated LDL cholesterol, directly enhances thrombus formation, an effect attenuated by statin therapy [53]. Smoking and obesity further amplify arterial risk through platelet activation, increased fibrinogen levels, chronic low-grade inflammation, and metabolic dysregulation [52,53,54]. Epidemiological data show that most patients experiencing arterial thrombotic events have at least one traditional risk factor, often in combination, and these factors are strongly associated with atherosclerotic disease across multiple vascular beds [54,55,56]. Notably, hypertension, diabetes, hyperlipidemia, and smoking are primarily linked to arterial, but not venous, thrombosis, reflecting fundamental differences in pathophysiology [52,57]. However, despite a lower prevalence of these classic risk factors, patients with inflammatory bowel disease remain at increased risk for arterial events, suggesting that their contribution is attenuated in IBD and that disease-specific inflammatory mechanisms play a dominant role [22,58,59].

#### 3.3.3. Conventional Systemic Therapies

Systemic corticosteroid therapy in IBD is consistently associated with a substantially increased risk of arterial thrombosis, including ACS and other major adverse cardiovascular events. Large population-based cohort studies demonstrate that IBD patients exposed to systemic corticosteroids have a markedly higher risk of ACS compared with both non-IBD controls and corticosteroid-unexposed IBD patients, with adjusted hazard ratios exceeding 5, an association that persists after adjustment for traditional cardiovascular risk factors and supports a direct prothrombotic effect of corticosteroids in this population [21]. These adverse vascular effects are multifactorial, encompassing the promotion of hypertension, dyslipidemia, insulin resistance, endothelial dysfunction, and direct prothrombotic activity. Accordingly, clinical guidelines and expert reviews consistently emphasize that prolonged corticosteroid exposure correlates with adverse cardiovascular outcomes and recommend minimizing steroid use in favor of corticosteroid-sparing therapies, particularly biologics, which are associated with lower rates of major adverse cardiovascular events [16,60,61,62]. The thrombotic risk appears to be especially pronounced in older patients and with longer treatment duration, underscoring the importance of restricting corticosteroid use to short-term induction of remission and transitioning promptly to maintenance therapy with alternative agents [60,62]. In contrast, 5-aminosalicylates (5-ASAs), including mesalamine and sulfasalazine, have a well-established safety profile and are widely used for induction and maintenance of remission in mild to moderate ulcerative colitis, particularly in older patients due to their lack of systemic immunosuppression [63]. Although their efficacy in Crohn’s disease is limited, there is no evidence from randomized trials, large observational studies, or clinical guidelines that 5-ASA therapy increases the risk of arterial thrombosis in IBD [60,63,64,65].

On the contrary, mechanistic data suggest a potential antithrombotic effect, as 5-ASA has been shown to inhibit platelet activation both in vitro and in vivo, with significantly lower ex vivo platelet activation observed in patients receiving oral 5-ASA [66]. While these findings suggest a theoretical protective effect against arterial thrombosis, direct clinical evidence demonstrating a reduction in cardiovascular events is currently lacking. Importantly, despite rare idiosyncratic adverse effects such as interstitial nephritis, pancreatitis, pericarditis, myocarditis, and pneumonitis, 5-ASAs have no established association with increased arterial thrombotic risk [63,65,67]. Thiopurines, including azathioprine and 6-mercaptopurine, occupy an intermediate position with respect to arterial thrombosis risk. Large nationwide cohort studies indicate that thiopurine exposure does not significantly reduce the risk of first acute arterial events in IBD patients (HR 0.93, 95% CI 0.82–1.05) [68]. However, among patients with a prior history of arterial thrombosis, thiopurine use has been associated with a modest but statistically significant reduction in recurrent events (HR 0.76, 95% CI 0.66–0.88), suggesting a potential role in secondary prevention, likely mediated through attenuation of systemic inflammation [69]. Notably, thiopurines have not been linked to an increased risk of arterial thrombosis, and their well-established long-term safety concerns, most prominently lymphoma and non-melanoma skin cancer, do not include arterial cardiovascular events [70,71,72,73]. Methotrexate represents an additional steroid-sparing immunomodulator, primarily used in Crohn’s disease, particularly in steroid-dependent or steroid-refractory patients, and as combination therapy with anti-TNF agents to reduce immunogenicity; however, it lacks efficacy in ulcerative colitis and is not recommended for this indication in current guidelines [60,74,75,76]. The relationship between methotrexate and arterial cardiovascular risk has been extensively studied in chronic inflammatory diseases. Observational studies and meta-analyses suggest that methotrexate use is associated with a reduced risk of total cardiovascular disease and myocardial infarction, likely reflecting the impact of effective inflammation control on vascular risk [77]. In contrast, the randomized CIRT trial in patients with stable atherosclerosis but without active systemic inflammation demonstrated no reduction in major adverse cardiovascular events with low-dose methotrexate compared with placebo (HR 0.96, 95% CI 0.79–1.16), indicating that any cardiovascular benefit may be context-dependent and confined to populations with high inflammatory burden [78]. Importantly, there is no evidence from randomized trials, meta-analyses, or systematic reviews to suggest that methotrexate increases the risk of arterial thrombosis in IBD or other inflammatory conditions, and its known toxicities do not include arterial thrombotic events [60,74,75,76,77,78].

#### 3.3.4. Biologic and Targeted Small-Molecule Therapies

Arterial cardiovascular events are largely driven by chronic systemic inflammation and the pro-atherogenic effects of tumor necrosis factor-α (TNF-α), a key mediator of endothelial dysfunction, plaque instability, and thrombosis [79]. Consistent with this mechanistic link, multiple large cohort studies and meta-analyses demonstrate that anti–TNF-α therapy is associated with a reduced risk of acute arterial events in IBD. In a nationwide French cohort, anti-TNF-α exposure was independently associated with a lower risk of incident arterial events (HR 0.79, 95% CI 0.6–0.95), with the greatest benefit observed in men with Crohn’s disease [68], and was also associated with reduced recurrence among patients with prior arterial events (HR 0.75, 95% CI 0.63–0.90) [69]. These findings are supported by meta-analytic data across immune-mediated inflammatory diseases, showing a significant reduction in major adverse cardiovascular events (MACE) with TNF inhibitors compared with conventional non-biologic therapies (HR 0.74, 95% CI 0.58–0.95), suggesting that effective suppression of systemic inflammation translates into vascular protection [68,79]. Importantly, anti-TNF-α therapy does not increase arterial thrombotic risk in IBD and may be protective, although class-specific risks such as opportunistic infections and heart failure must be considered [68,69,79,80].

Vedolizumab, a gut-selective α4β7 integrin antagonist, has not been associated with an increased risk of arterial thrombosis. Integrated safety analyses, long-term observational cohorts, and narrative reviews consistently report no excess incidence of myocardial infarction or stroke among vedolizumab-treated patients compared with other biologics or standard care [81,82,83]. Although hypothesis-generating pharmacovigilance analyses have reported cardiovascular signals, these findings have not been corroborated in controlled trials or robust real-world cohorts and are likely influenced by reporting bias and confounding [84]. Overall, current evidence supports a favorable cardiovascular safety profile consistent with vedolizumab’s gut-selective mechanism [81,82,83].

Similarly, available data do not demonstrate a consistent increase in arterial thrombosis or MACE associated with ustekinumab, an inhibitor of IL-12 and IL-23-mediated signaling, in IBD or other immune-mediated inflammatory diseases. Large observational cohorts and postauthorization safety studies in psoriasis and psoriatic arthritis populations report no significant difference in MACE incidence compared with TNF inhibitors or other biologics, with hazard ratios close to unity [85,86], findings supported by integrated analyses of phase II/III trials and long-term follow-up data [87,88]. However, some studies have raised concerns regarding a potential early increase in severe cardiovascular events among patients with high baseline cardiovascular risk. A large case-time-control study identified an association between ustekinumab initiation and acute coronary syndrome or stroke in high-risk individuals (OR 4.17, 95% CI 1.19–14.59), but not in low-risk patients [89]. Meta-analyses using the Peto method [90] have also suggested a possible signal for IL-12/23 antagonists, although these findings are inconsistent across analytical approaches and limited by low event rate. Taken together, the totality of evidence does not support a general increase in arterial thrombotic risk with ustekinumab, though caution may be warranted in patients with substantial baseline cardiovascular risk, particularly early after treatment initiation.

Janus kinase (JAK) inhibitors, including tofacitinib, upadacitinib, and filgotinib, are effective therapies for moderate-to-severe ulcerative colitis, with upadacitinib also approved for Crohn’s disease [67,74,91]. Despite cardiovascular safety concerns arising from other inflammatory disease populations, systematic reviews and meta-analyses of randomized controlled trials have not demonstrated a statistically significant increase in arterial thrombosis or MACE with JAK inhibitors compared with placebo or active comparators. In a meta-analysis of more than 19,000 patients, the pooled odds ratio for arterial thrombosis was 0.82 (95% CI 0.43–1.56), with no significant differences by agent or dose [92]. Network meta-analyses in IBD similarly found no significant increase in cardiovascular events, although non-significant trends toward higher risk at supratherapeutic doses were observed [93], and observational comparisons with TNF antagonists showed no difference in MACE risk (HR 0.50, 95% CI 0.19–1.30) [94]. Nonetheless, regulatory agencies and clinical guidelines recommend caution in patients with high baseline cardiovascular risk, based on findings from the ORAL Surveillance trial in rheumatoid arthritis, where tofacitinib, particularly at higher doses, was associated with increased MACE in older patients with cardiovascular risk factors [91,92,93]. While this signal has not been reproduced in IBD populations, careful risk stratification is advised [91,92].

Sphingosine-1-phosphate (S1P) receptor modulators, including ozanimod and etrasimod, are approved for the treatment of moderately to severely active ulcerative colitis and have demonstrated efficacy in randomized trials and meta-analyses [95]. Their cardiovascular safety profile is characterized primarily by transient bradycardia, atrioventricular conduction abnormalities, and a possible increase in hypertension, effects that are mechanistically related to S1P receptor signaling in cardiac tissue and are generally dose-dependent and reversible [96,97,98]. Importantly, current evidence from systematic reviews, meta-analyses, and consensus guidelines does not indicate an increased risk of arterial thrombosis or MACE associated with S1P modulators in IBD [95,96,97,98]. While cardiovascular assessment prior to initiation is recommended, particularly in patients with pre-existing cardiac disease, available data suggest a low risk of arterial thrombotic events, although long-term real-world safety data remain limited [62,67,95,96,97,98]. Collectively, available evidence highlights marked heterogeneity in arterial thrombotic risk across IBD therapies, with key differences summarized in Table 3.

#### 3.3.5. Inherited and Acquired Thrombophillia

The relative contributions of inherited versus acquired thrombophilic factors to arterial thrombosis in patients with IBD differ in important and clinically relevant ways. While classical inherited thrombophilias are well-established contributors to venous thromboembolism, accumulating evidence indicates that arterial thrombotic events in IBD are driven predominantly by acquired, inflammation-related mechanisms rather than by genetic predisposition alone. Inherited thrombophilias such as factor V Leiden and the prothrombin G20210A mutation do confer an increased overall thrombotic risk in patients with IBD, with monogenic and polygenic risk factors exerting additive effects [99,100,101,102]. However, most studies investigating thrombosis in IBD have focused on venous events, and data specifically addressing arterial thrombosis consistently demonstrate that the prevalence of major inherited thrombophilias among IBD patients with arterial events is lower than that observed in non-IBD individuals with comparable vascular disease [103]. These observations support the concept that inherited thrombophilia plays a secondary, contributory role rather than a primary causal role in arterial thrombogenesis in IBD. Within this context, genetic polymorphisms involving MTHFR and PAI-1 are frequently identified and often raise concerns regarding their clinical significance. MTHFR variants, most notably C677T and A1298C, are associated with reduced enzymatic activity and may lead to mild hyperhomocysteinemia under specific conditions such as folate deficiency [104]. Nevertheless, robust evidence has demonstrated that MTHFR genotypes themselves do not constitute an independent thrombotic risk factor. Accordingly, a recent international call to action has explicitly recommended against the inclusion of MTHFR genotyping in thrombophilia screening, emphasizing that such testing does not inform clinical management, increases patient anxiety, and unnecessarily increases healthcare costs [104]. Similar considerations apply to PAI-1 promoter polymorphisms, including the 4G/5G variant, which are not recognized as validated thrombophilic risk factors [105,106]. Importantly, both MTHFR and PAI-1 4G/5G genotypes are highly prevalent in the general population, with frequencies approaching 25–45% among healthy individuals [107,108]. Except for its association with mild hyperhomocysteinemia, the MTHFR C677T mutation has been reported as a risk factor for arterial thrombosis in Chinese Han patients with antiphospholipid syndrome (APS) [109]. Similarly, the presence of the 4G allele of the PAI-1 4G/5G polymorphism may represent an additional risk factor for arterial thrombosis in APS [110]. However, testing for MTHFR gene mutations in patients with positive antiphospholipid antibodies (aPL) is not recommended, as MTHFR polymorphisms do not confer an increased risk of venous thromboembolism (VTE) or adverse pregnancy outcomes, and their assessment does not influence clinical management in this context [111,112]. Interestingly, some studies suggest that combinations of heritable thrombophilic genotypes, such as PAI-1 4G/4G in conjunction with other mutations (e.g., factor V Leiden or prothrombin G20210A), may be more frequent in patients with definite antiphospholipid syndrome and could help identify aPL-positive individuals at higher risk for clinical manifestations [113]. Nevertheless, current guidelines do not recommend routine PAI-1 mutation testing for thrombophilia evaluation. Consequently, the presence of these variants should not be interpreted as causal explanations for thrombotic events, particularly arterial thrombosis in IBD.

In contrast, acquired prothrombotic abnormalities appear to represent the potential drivers of arterial thrombosis in this population, with active intestinal and systemic inflammation serving as the central pathogenic trigger [114,115,116]. Among these acquired factors, antiphospholipid antibodies, particularly anticardiolipin antibodies, and elevations in procoagulant factors such as factor VIII have been documented in both adult and pediatric patients with IBD who experience thromboembolic complications, including arterial events [114,115]. In addition, patients with IBD frequently exhibit increased plasma levels of fibrinogen, factor VII, and lipoprotein(a), all of which are recognized contributors to arterial occlusive disease and are found at higher concentrations in IBD compared with healthy controls [116]. Hyperhomocysteinemia represents another clinically relevant acquired thrombophilic state in IBD and provides an important mechanistic link between chronic inflammation, nutritional deficiencies, and vascular pathology. Homocysteinemia refers to elevated plasma homocysteine, a sulfur-containing amino acid generated during methionine metabolism, with hyperhomocysteinemia commonly defined as plasma levels exceeding 15 µmol/L and substantially increased thrombotic risk at concentrations above 30 µmol/L [117,118]. The most frequent causes include deficiencies of folate, vitamin B12, and vitamin B6, renal impairment, and inherited defects in homocysteine metabolism, all of which may be encountered in patients with IBD due to malabsorption, chronic inflammation, or systemic disease burden [118,119,120]. Elevated homocysteine is a well-established risk factor for arterial thrombosis, including stroke, myocardial infarction, and peripheral arterial disease, primarily through its deleterious effects on vascular biology [117,118,121,122,123,124]. Mechanistically, homocysteine promotes endothelial dysfunction, oxidative stress, and inflammatory signaling, thereby accelerating atherogenesis and creating a prothrombotic vascular milieu [118,121,122]. In addition, homocysteine and its reactive metabolites, particularly homocysteine-thiolactone, can induce homocysteinylation of plasma proteins such as fibrinogen. This post-translational modification alters fibrin architecture, resulting in denser, less permeable fibrin clots that are more resistant to fibrinolysis and thus more likely to persist and propagate arterial thrombosis. Homocysteinylated proteins further acquire proinflammatory and procoagulant properties, amplifying vascular injury [121,124,125]. Epidemiological studies demonstrate a clear dose-dependent relationship between plasma homocysteine levels and arterial thrombotic events. Meta-analyses indicate that a 3 µmol/L increase in homocysteine concentration is associated with approximately a 19% increase in stroke risk, while elevated homocysteine levels have been shown to double the risk of significant carotid artery stenosis in elderly populations [123]. Severe hyperhomocysteinemia is associated with premature vascular disease and increased mortality [118,121,124]. However, the clinical relevance of moderate homocysteine elevations remains a matter of debate, as randomized trials of homocysteine-lowering strategies, such as B vitamin supplementation, have not consistently demonstrated reductions in cardiovascular events, although some meta-analyses suggest a modest protective effect against stroke [118,123].

### 3.4. Case-Specific Clinical Commentary

Our case highlights the intricate relationship between severe UC, systemic inflammation, and a tendency toward thrombosis, culminating in widespread arterial thrombosis in a middle-aged male patient. While VTE is well recognized in cases of UC, arterial events are significantly rarer but can be exceedingly dangerous, emphasizing the importance of careful monitoring during acute exacerbations.

#### 3.4.1. Diagnostic Evaluation

The diagnostic workup was guided by contemporary recommendations for the evaluation of unexplained arterial thrombosis [126], emphasizing a structured and exclusion-based approach. Accordingly, a comprehensive assessment was undertaken to systematically evaluate atherosclerotic disease, potential cardioembolic sources, systemic and autoimmune disorders, infectious etiologies, medication-related prothrombotic factors, vascular abnormalities, and inherited or acquired thrombophilias. Traditional cardiovascular risk factors were notably absent, and multimodal imaging did not demonstrate significant atherosclerotic disease. Computed tomography angiography identified a mural thrombus in the ascending aorta, which was considered the most plausible embolic source accounting for the renal, splenic, and hepatic infarctions. Concomitantly, alternative embolic sources were carefully excluded. Electrocardiography and transthoracic echocardiography revealed no atrial arrhythmias, intracardiac thrombi, or valvular pathology, while there was no evidence of patent foramen ovale. Carotid Doppler ultrasonography did not show hemodynamically relevant stenosis, and venous imaging was negative, arguing against paradoxical embolism. An extensive laboratory evaluation was performed to exclude systemic autoimmune disease and vasculitis, including RF, anti CCP, ANA, pANCA, cANCA, and anti-GBM, all of which were negative. Infectious causes associated with arterial thrombosis [126] were also excluded, with negative testing for hepatitis B surface antigen, hepatitis C virus, HIV, and SARS-CoV-2. Screening for inherited and acquired thrombophilia, including antiphospholipid antibodies, protein C and protein S activity, antithrombin levels, factor V Leiden, and the prothrombin G20210A mutation, yielded no clinically significant abnormalities. There were no laboratory features suggestive of hemolysis or cytopenias, rendering further evaluation for paroxysmal nocturnal hemoglobinuria or myeloproliferative neoplasms unnecessary. In addition, tumor markers were unremarkable, arguing against an occult malignancy-associated hypercoagulable state [127]. A careful medication history excluded exposure to agents known to increase arterial thrombotic risk, including erythropoietin, oral contraceptives, anti-angiogenic therapies (e.g., bevacizumab), selective estrogen receptor modulators, protease inhibitors, gonadotropin-releasing hormone agonists, tyrosine kinase inhibitors, and antipsychotic drugs [126,128]. The only abnormalities identified were homozygosity for methylenetetrahydrofolate reductase polymorphisms and the presence of the PAI-1 4G/5G genotype, accompanied by mild hyperhomocysteinemia. Importantly, based on current evidence, these findings are not considered independent or causative risk factors for arterial thrombosis and are highly prevalent in the general population [104,105,106,107,108,118,123]. As such, they were interpreted as incidental or, at most, background modifiers rather than explanatory determinants of the patient’s thrombotic presentation.

#### 3.4.2. Acute Phase Management

After exclusion of alternative etiologies and identification of aortic mural thrombosis as the most likely embolic source, therapeutic management was directed toward controlling both the underlying intestinal inflammation and the associated thrombotic process. The patient was treated for acute severe ulcerative colitis with intravenous corticosteroids and fluid resuscitation, high-dose oral mesalazine, and intravenous metronidazole, together with prophylactic low-molecular-weight heparin, in accordance with ECCO recommendations mandating routine thromboprophylaxis in hospitalized patients with active disease [129]. In line with established treatment algorithms, clinical response to intravenous corticosteroids was assessed on day three. Given a favorable response, escalation to rescue therapy with infliximab or ciclosporin was not required, and surgical intervention was not indicated.

In the setting of acute neurological deterioration accompanied by imaging evidence of ascending aortic mural thrombosis with systemic embolization, systemic anticoagulation was selected as the cornerstone of initial management. UFH was chosen as the initial anticoagulant strategy based on context-specific clinical considerations rather than as a universally preferred agent for arterial thrombosis. The optimal management of aortic mural thrombus remains incompletely standardized and depends on thrombus morphology (size, mobility, and location), the presence of underlying aortic pathology, embolic burden, and patient-specific comorbidities [130,131]. According to 2022 ACC/AHA guidelines [131], asymptomatic secondary mural thrombi are often managed conservatively, whereas patients presenting with embolic events require individualized treatment strategies that may include anticoagulation, endovascular intervention, or open surgery. Anticoagulation is frequently used as first-line therapy, particularly when invasive approaches are not feasible or carry excessive risk, although higher rates of thrombus persistence and recurrence have been reported in selected high-risk morphologies [131]. In the present case, UFH was favored over low-molecular-weight heparin (LMWH) because of its pharmacokinetic and pharmacodynamic properties, which are particularly advantageous in unstable clinical scenarios with a high and unpredictable bleeding risk [132,133]. UFH has a short plasma half-life of approximately 1–2 h and can be fully reversed with protamine sulfate, allowing rapid interruption of anticoagulation in the event of hemorrhage or clinical deterioration. In contrast, LMWH has a longer half-life, is only partially reversible, and is predominantly renally cleared, limiting its safety margin in patients with fluctuating renal function or systemic inflammation [132,133]. In patients with severe active ulcerative colitis, such as in this case, the baseline risk of gastrointestinal bleeding is substantially increased, rendering anticoagulants with prolonged or less controllable anticoagulant effects less suitable during the acute phase [134]. At the same time, the limitations of UFH must be acknowledged. Its use requires continuous intravenous administration and frequent laboratory monitoring, most commonly with activated partial thromboplastin time, which is not fully standardized and may exhibit significant inter- and intra-laboratory variability. UFH dosing is therefore highly operator-dependent and may be influenced by acute-phase reactants, fluctuations in coagulation factor levels, and changes in plasma proteins, leading to variable anticoagulant responses. In addition, UFH carries a recognized risk of heparin-induced thrombocytopenia and requires close hematological surveillance. These drawbacks limit its practicality outside closely monitored settings and preclude its routine use as a default anticoagulant for arterial thrombosis [135,136,137]. Nevertheless, in the acute phase of severe inflammatory disease with dynamic thrombotic and bleeding risks, the reversibility and titratability of UFH outweigh these limitations.

Invasive strategies for thrombus exclusion were carefully considered but not pursued. Surgical or endovascular intervention is associated with lower recurrence rates in patients with large, mobile, or pedunculated thrombi and in those with recurrent embolization despite anticoagulation; however, these approaches require anatomical suitability and clinical stability [131,138]. In this case, the thrombus was located in the ascending aorta, a region associated with high technical complexity and peri-procedural risk [139]. Moreover, the patient’s severe systemic inflammation and active ulcerative colitis significantly increased perioperative bleeding risk, rendering invasive intervention unfavorable [140]. Imaging did not demonstrate a highly mobile or enlarging thrombus mandating urgent exclusion, and no progression was observed under anticoagulation. Endovascular approaches, such as thoracic endovascular aortic repair, are increasingly favored for descending aortic mural thrombi but remain limited in ascending aortic involvement, while open surgery is generally reserved for refractory cases or those not amenable to endovascular repair [138,139,141,142,143,144].

Systemic thrombolysis was contraindicated because the patient presented outside the therapeutic time window and neuroimaging demonstrated established ischemic lesions rather than an acute large-vessel occlusion amenable to reperfusion [145]. Furthermore, active mucosal bleeding from severe ulcerative colitis constituted a major contraindication due to the unacceptably high risk of life-threatening gastrointestinal hemorrhage [146]. Mechanical thrombectomy was similarly not indicated, as neuroimaging did not reveal a proximal intracranial large-vessel occlusion suitable for endovascular intervention, and the infarct pattern was consistent with multifocal embolization rather than a single targetable lesion [147].

Taken together, therapeutic UFH administered under close multidisciplinary supervision represented the most balanced and adaptable strategy in this clinical context, allowing effective suppression of ongoing embolization while preserving the flexibility required to rapidly respond to bleeding complications. Complete radiological resolution of the aortic and iliac thrombi was achieved without hemorrhagic complications, while established visceral infarctions remained stable. This management approach should be interpreted as an individualized, context-driven decision rather than a general endorsement of UFH for arterial thrombosis, underscoring the importance of tailoring anticoagulation and interventional strategies to thrombus characteristics, inflammatory activity, bleeding risk, and anatomical feasibility in patients with inflammatory bowel disease.

#### 3.4.3. Remission Maintenance Strategy

Current international guidelines, including the ECCO 2022 [129], AGA 2024 [67], and British Society of Gastroenterology 2025 [148] recommendations, endorse several advanced therapies as appropriate first-line options for biologic-naive patients with moderate to severe ulcerative colitis. These include TNF-α antagonists, vedolizumab, ustekinumab, S1P receptor modulators, and IL-23 inhibitors. Within this framework, treatment selection should be individualized, balancing efficacy and safety considerations, particularly in patients with relevant comorbidities. In the present case of a biologic-naive patient with severe ulcerative colitis complicated by arterial thrombosis, vedolizumab was selected as the initial biologic therapy following a careful risk–benefit assessment. From an efficacy perspective, robust evidence demonstrates that vedolizumab is at least comparable, and in several analyses superior, to TNF-α antagonists in biologic-naive populations [80,98,149,150,151,152]. Head-to-head and network meta-analyses consistently demonstrate that vedolizumab is at least as effective, and in some analyses superior, to TNF-α antagonists for induction and maintenance of clinical remission and endoscopic improvement in biologic-naive patients. The VARSITY trial, a prospective, randomized, head-to-head study, showed that vedolizumab was superior to adalimumab for both clinical remission and endoscopic improvement [80,98,149]. Network meta-analyses further support vedolizumab’s superiority over adalimumab for maintenance of clinical remission and endoscopic improvement, with vedolizumab ranking highest among available biologics for these outcomes [80,149]. Real-world retrospective studies also report higher rates of clinical remission, steroid-free remission, and drug persistence with vedolizumab compared to infliximab and other TNF-α antagonists in biologic-naive cohorts [150,151,152]. Mechanistically, vedolizumab is a humanized monoclonal antibody that targets the α4β7 integrin, selectively inhibiting its interaction with mucosal addressing cell adhesion molecule-1 (MAdCAM-1). This gut-selective mechanism blocks lymphocyte trafficking to the intestinal mucosa, resulting in potent local anti-inflammatory activity while limiting systemic immunosuppression [153,154]. As a consequence of this targeted mode of action, vedolizumab is associated with a favorable systemic safety profile, an aspect of particular importance in patients with recent vascular complications. Consistent with this mechanistic rationale, large real-world cohorts and network meta-analyses have demonstrated that vedolizumab is associated with lower rates of serious adverse events and serious infections compared with TNF-α antagonists [98,150,152,155]. This safety advantage is especially relevant in patients with increased susceptibility to complications due to comorbid conditions [67]. In both direct and indirect comparisons, vedolizumab consistently ranks among the safest advanced therapies for ulcerative colitis [98,155]. Although differences in efficacy between vedolizumab and infliximab may be less pronounced than those observed with adalimumab, real-world data suggest higher treatment persistence and lower rates of primary non-response with vedolizumab [151]. Overall, the available evidence supports vedolizumab as a highly effective first-line biologic option in biologic-naive ulcerative colitis, offering efficacy comparable to or exceeding that of TNF-α antagonists while maintaining a favorable long-term safety profile.

#### 3.4.4. Secondary Thrombosis Prevention

The thrombotic events in this patient were interpreted as the consequence of a transient, inflammation-driven prothrombotic state associated with severe active ulcerative colitis rather than a persistent thrombophilia. Extensive diagnostic evaluation excluded secondary causes of arterial thrombosis. The distribution of thrombotic events was predominantly arterial, suggesting that platelet activation and endothelial dysfunction driven by inflammation were the principal pathophysiological mechanisms. On this basis, antiplatelet therapy was considered a rational strategy for secondary prevention. Although the hypercoagulable state was presumed to be transient and inflammation-driven, the occurrence of ischemic stroke confers an inherently increased risk of recurrence, and current consensus supports ongoing secondary prevention unless the event can be unequivocally attributed to a fully reversible, nonrecurring cause without residual risk. In this context, indefinite antiplatelet therapy is generally recommended for secondary prevention after non-cardioembolic, non-atherosclerotic ischemic stroke, even when the initial trigger is transient [156,157,158,159]. Periodic reassessment of thrombotic risk and inflammatory disease activity is therefore essential to determine the long-term need for continued therapy.

Clopidogrel monotherapy was selected as the most appropriate antiplatelet agent, balancing efficacy and safety. From an efficacy standpoint, robust evidence supports the superiority of clopidogrel over aspirin for secondary prevention of atherothrombotic events, including coronary artery disease, ischemic stroke, and peripheral artery disease, as demonstrated in multiple meta-analyses and the landmark CAPRIE trial [160,161,162]. In line with this evidence, the European Society of Cardiology (ESC) Guidelines on the Diagnosis and Treatment of Peripheral Arterial Diseases recommend clopidogrel over aspirin for long-term secondary prevention, further supporting its use in a patient with documented arterial thrombosis [163]. Moreover, from a safety perspective, clopidogrel monotherapy is consistently associated with a lower risk of major gastrointestinal bleeding compared with oral anticoagulant therapy, including both vitamin K antagonists and most direct oral anticoagulants (DOACs). Reported annual rates of major gastrointestinal bleeding with clopidogrel range from approximately 0.85% to 1.6%, compared with about 2.5% per year with warfarin, and similar or lower than those seen with DOACs, depending on the specific agent [164,165]. Certain direct oral anticoagulants, particularly dabigatran and rivaroxaban, are associated with gastrointestinal bleeding risks comparable to or exceeding those of warfarin, while apixaban appears safer but does not demonstrate a clear bleeding advantage over clopidogrel [165]. Additionally, comparative data from pooled analyses and case–control studies consistently show that clopidogrel has a lower incidence of major gastrointestinal bleeding than warfarin and similar or slightly lower rates than aspirin [164,166]. Specifically, the risk of hospitalization for GI bleeding is 0.7% with clopidogrel versus 2.5% with warfarin [164]. The risk with DOACs is variable, but overall, the risk of lower GI bleeding is similar between DOACs and conventional anticoagulants, and there is no evidence that DOACs confer a lower GI bleeding risk than clopidogrel [165].

Overall, the risk of hospitalization for gastrointestinal bleeding is substantially lower with clopidogrel than with systemic anticoagulation [164,165], a consideration of particular importance in patients with recent severe inflammatory bowel disease activity. Accordingly, clopidogrel was initiated as a secondary prevention strategy, intended to reduce the risk of recurrent arterial thrombosis while minimizing bleeding complications during the period of active systemic inflammation. Ongoing therapy was planned to be reassessed after sustained clinical, biochemical, and endoscopic remission of ulcerative colitis was achieved, with strict control of intestinal inflammation regarded as the cornerstone of long-term thrombotic risk reduction.

#### 3.4.5. Adjunctive and Supportive Therapy

In addition to the previously described therapies, targeted supportive interventions were implemented. Mild hyperhomocysteinemia was managed with folic acid, vitamin B6, and vitamin B12 supplementation [41]. Iron deficiency anemia was treated in accordance with current guidelines, which recommend oral iron as first-line therapy in patients with mild anemia (hemoglobin ≥ 11 g/dL), inactive or mildly active disease, and no prior intolerance [98]. However, conventional oral iron formulations are frequently limited by gastrointestinal side effects and poor adherence. To overcome these limitations, liposomal iron was selected. This novel oral formulation is designed to improve intestinal absorption and systemic bioavailability while minimizing gastrointestinal intolerance, thereby enhancing treatment adherence [99]. Our recent data further support this approach, demonstrating that liposomal iron effectively corrected anemia with excellent tolerability, underscoring its potential as a preferred option for iron replacement in patients with inflammatory bowel disease [100]. At six months of follow-up, the patient remained in clinical, biochemical, and endoscopic remission, with no recurrent thrombotic events. This structured diagnostic and therapeutic strategy provides a framework for interpreting the present case and, when integrated with current literature on inflammatory bowel disease–associated thrombosis, highlights both shared pathophysiological mechanisms and the distinctive features of this presentation.

## 4. Future Directions

Several important research priorities arise from this case and the existing literature. First, we need well-designed, multicenter studies with enough participants to accurately define the true incidence and clinical spectrum of arterial thrombosis across IBD subtypes. These studies should measure the risk during flares and remission and identify which biomarkers, such as CRP levels, platelet counts, and markers of endothelial activation, best predict these events. Second, we should conduct studies to understand how intestinal inflammation leads to systemic endothelial damage and increased platelet activity. It is also crucial to explore whether specific genetic variants and metabolic factors, like issues with the folate-homocysteine pathway, influence the risk of thrombosis in a significant way. These efforts should go hand in hand with creating and testing practical tools to assess risk that can be used at the bedside during hospital stays and shortly after discharge. Third, we need research comparing different strategies for secondary prevention after arterial events in IBD. This includes direct comparisons of antiplatelet and anticoagulant treatments, the length of therapy, and the risks of bleeding in cases of active or recently active colitis. Finally, we need to further clarify the safety of specific therapies concerning vascular health. Large, real-world studies and safety monitoring programs should systematically compare new IBD treatments regarding arterial outcomes and serious cardiovascular events, especially in patients who have had previous thrombosis or who are at high risk for cardiovascular issues. Addressing these gaps in knowledge is vital for shifting from reactive care to preventive, personalized vascular health in IBD.

## 5. Conclusions

Diffuse arterial thrombosis is an uncommon but life-threatening complication of severe ulcerative colitis that may occur even in the absence of traditional cardiovascular risk factors. The present case illustrates a clinically plausible model of inflammation-driven immunothrombosis in which acute inflammatory burden serves as the primary trigger, while additional modifiers may potentiate risk rather than act as independent causes. Beyond documenting a rare clinical phenotype, this report underscores several practice-relevant considerations: heightened vigilance for arterial events during periods of severe disease activity, the necessity of systematically excluding alternative etiologies to avoid diagnostic anchoring, and the importance of prioritizing sustained inflammatory control with therapies whose systemic safety profiles are appropriate for patients with increased vascular vulnerability. Taken together, these observations highlight the need for an individualized, risk-adapted clinical framework that integrates disease activity, clinical context, and comorbidity burden when managing complex presentations of ulcerative colitis. Given the inherent limitations of inference from a single case, the concepts outlined herein should be regarded as hypothesis-generating and intended to inform future research rather than to establish definitive management recommendations.

## Figures and Tables

**Table 1:** Laboratory data during hospitalisation and outpatient follow-up.

Variable	Initial (Day 0)	Day 4	Day 12	After Six Months	Reference Range, Adults
Leukocytes	17.3 × 10^9^/L	13.7 × 10^9^/L	7.2 × 10^9^/L	7.8 × 10^9^/L	4–10 × 10^9^/L
Hemoglobin	107 g/L	105 g/L	115 g/L	144 g/L	138–175 g/L
MCV	72 fL	75 fL	75 fL	92 fL	83.0–100.0 fL
Platelets	576 × 10^9^/L	530 × 10^9^/L	380 × 10^9^/L	327 × 10^9^/L	150–450 × 10^9^/L
Ferittin	40 ng/mL	/	/	310 ng/mL	30–400 ng/mL
TSAT	13%	/	/	31%	20–50%
CRP	73.6 mg/L	36.7 mg/L	8.4 mg/L	4.4 mg/L	<5.0 mg/L
D-dimer	3.9 mg/L	4.29 mg/L	2.8 mg/L	/	<5.0 mg/L FEU
Fibrinogen	6.4 g/L	5.5 g/L	4.1 g/L	/	2.0–4.0 g/L
Homocystein	21.4 µmol/L	/	/	9.8 µmol/L	5–15 µmol/L
CEA, CA 19-9, NSE, CA 72-4, AFP, PSA free, PSA total,	/	Negative	/	/	Negative
HbsAg, HCV, HIV, SARS-CoV-2	/	Negative	/	/	Negative
RF, anti-CCP, ANA	/	Negative	/	/	Negative
cANCA, pANCA, anti-GBM	/	Negative	/	/	Negative
LAC ACA IgM/IgG Anti-β2 GLP IgM IgG Ab	/	Negative	/	/	Negative
Protein C	/	90%	/	/	60–130%
Protein S	/	93%	/	/	60–130%
Antithrombin	/	97%	/	/	80–120%
factor V Leiden prothrombin G20210A	/	Negative	/	/	Negative
MTHFR C677T	/	Positive	/	/	Negative
PAI-1 4G/5G	/	Positive	/	/	Negative

**Abbreviations**: **MCV**, mean corpuscular volume; **TSAT**, transferrin saturation; **CRP**, C-reactive protein; **CEA**, carcinoembryonic antigen; **CA 19-9**, carbohydrate antigen; **NSE**, neuron-specific enolase; **CA 72-4**, cancer antigen; **AFP**, alpha-fetoprotein; **PSA**, prostate-specific antigen; **HBsAg**, hepatitis B surface antigen; **HCV**, hepatitis C virus; **HIV**, human immunodeficiency virus; **SARS-CoV-2**, severe acute respiratory syndrome Coronavirus 2; **RF**, reuma factor; **anti-CCP**, anti-cyclic citrulinated peptide; **ANA**, antinuclear antibodies; **cANCA**, cytoplasmatic anti-neutrophil cytoplasmic antibodies; **pANCA**, perinuclear anti-neutrophil cytoplasmic antibodies; **anti-GBM**, anti-glomerular basement membrane; **LAC**, lupus anticoagulant; **ACA**, anti-cardiolipin antibodies; **anti-β2 GLP**, anti-glycoprotein; **MTHFR**, methylenetetrahydrofolate reductase; **PAI-1**, plasminogen activator inhibitor-1 4G/5G polymorphism.

**Table 3:** Arterial Thrombotic Risk Associated with IBD Therapies.

Therapy Class	Effect on Arterial Thrombosis	Key Evidence	Clinical Interpretation
Systemic corticosteroids	Increased risk	Population-based cohorts; HR > 5 for ACS	Major prothrombotic signal; restrict to short-term induction
5-ASA	Neutral/possibly protective	No increased risk; platelet inhibition in mechanistic studies	Arterially safe; no proven clinical CV protection
Thiopurines	Neutral (primary)/reduced recurrence	Nationwide cohorts; HR 0.76 for recurrent events	Possible secondary prevention benefit
Methotrexate	Neutral; context-dependent benefit	Observational benefit; CIRT trial neutral	No arterial harm; benefit likely limited to high inflammatory burden
Anti-TNF agents	Reduced risk	Large cohorts and meta-analyses; HR ~0.75–0.80	Most consistent arterial protective signal
Integrin antagonist	Neutral	Integrated safety analyses; no MI or stroke signal	Favorable CV safety; gut-selective
Anti-IL-12/23 agents	Neutral overall; early risk in high-risk patients	Cohorts and trials; case-time-control signal	Use caution early in patients with high CV risk
JAK inhibitors	Neutral in IBD	RCT meta-analyses; RA signal in ORAL Surveillance	Risk stratify; avoid high doses in high-risk patients
S1P receptor modulators	Neutral	RCTs and meta-analyses; no MACE signal	Monitor bradycardia and BP; low thrombotic risk

## Data Availability

This article, being a case report and review, does not contain any primary data for sharing. The data discussed are derived from previously published studies and the patient’s medical records.

## Abbreviations

ACS	Acute Coronary Syndrome
ACA	Anti-cardiolipin antibodies
aHR	Adjusted Hazard Ratio
AFP	Alpha-fetoprotein
ANA	Antinuclear Antibodies
ANCA	Antineutrophil Cytoplasmic Antibodies
anti-β2 GLP Ab	Anti-glycoprotein antibodies
anti-CCP	Anti-cyclic citrullinated peptide
anti-GBM Ab	Anti-glomerular basement membrane antibodies
AMI	Acute Myocardial Infarction
ATIII	Antithrombin III
ATED	Arterial Thromboembolic Disease
CA 19-9	Carbohydrate antigen 19-9
CA 72-4	Cancer antigen 72-4
CD	Crohn’s Disease
CD40	Cluster of Differentiation 40
CD40L	Cluster of Differentiation 40 Ligand
CEA	Carcinoembryonic antigen
CI	Confidence Interval
cANCA	Cytoplasmatic anti-neutrophil cytoplasmic antibodies
CRP	C-Reactive Protein
CVA	Cerebrovascular Accident
EIM	Extra-Intestinal Manifestations
ESC	European Society of Cardiology
F1+2	Prothrombin fragment 1+2
FV	Coagulation factor V
FVII	Coagulation factor VII
FVIII	Coagulation factor VIII
FXI	Coagulation factor XI
FXIII	Coagulation factor XIII
HBsAg	Hepatitis B surface antigen
HBV	Hepatitis B Virus
HCV	Hepatitis C Virus
HF	Heart Failure
HIV	Human Immunodeficiency Virus
HR	Hazard Ratio
IBD	Inflammatory Bowel Disease
IBD-U	Inflammatory Bowel Disease–Unclassified
ICAM-1	Intercellular Adhesion Molecule-1
IFN-γ	Interferon Gamma
IHD	Ischemic Heart Disease
IL-6	Interleukin-6
IRR	Incidence Rate Ratio
LAC	Lupus anticoagulant
LPS	Lipopolysaccharide
MCV	Mean corpuscular volume
MI	Myocardial Infarction
MPV	Mean Platelet Volume
MTHFR	Methylenetetrahydrofolate reductase
NIS	National Inpatient Sample
NSE	Neuron-specific enolase
OR	Odds Ratio
PAD	Peripheral Arterial Disease
PAI-1	Plasminogen Activator Inhibitor-1
pANCA	Perinuclear anti-neutrophil cytoplasmic antibodies
PSA	Prostate-specific antigen
RF	Rheumatoid factor
SARS-CoV-2	Severe acute respiratory syndrome Coronavirus 2
TAFI	Thrombin-Activatable Fibrinolysis Inhibitor
TAT	Thrombin–Antithrombin Complex
TFPI	Tissue Factor Pathway Inhibitor
TIA	Transient Ischemic Attack
TLR	Toll-like receptor
TNF-α	Tumor Necrosis Factor Alpha
t-PA	Tissue Plasminogen Activator
TSAT	Transferrin saturation
UC	Ulcerative Colitis
VCAM-1	Vascular Cell Adhesion Molecule-1
VEGF	Vascular endothelial growth factor
vWF	Von Willebrand factor
VTE	Venous Thromboembolism
